# The effects of sequencing depth on the assembly of coding and noncoding transcripts in the human genome

**DOI:** 10.1186/s12864-022-08717-z

**Published:** 2022-07-04

**Authors:** Isaac Adeyemi Babarinde, Andrew Paul Hutchins

**Affiliations:** Shenzhen Key Laboratory of Gene Regulation and Systems Biology, Department of Biology, School of Life Sciences, Southern University of Science and Technology, Shenzhen, 518055 China

**Keywords:** Transcript assembly, Sequencing depth, Coding transcripts, Noncoding transcripts, Transposable elements

## Abstract

Investigating the functions and activities of genes requires proper annotation of the transcribed units. However, transcript assembly efforts have produced a surprisingly large variation in the number of transcripts, and especially so for noncoding transcripts. This heterogeneity in assembled transcript sets might be partially explained by sequencing depth. Here, we used real and simulated short-read sequencing data as well as long-read data to systematically investigate the impact of sequencing depths on the accuracy of assembled transcripts. We assembled and analyzed transcripts from 671 human short-read data sets and four long-read data sets. At the first level, there is a positive correlation between the number of reads and the number of recovered transcripts. However, the effect of the sequencing depth varied based on cell or tissue type, the type of read and the nature and expression levels of the transcripts. The detection of coding transcripts saturated rapidly with both short and long-reads, however, there was no sign of early saturation for noncoding transcripts at any sequencing depth. Increasing long-read sequencing depth specifically benefited transcripts containing transposable elements. Finally, we show how single-cell RNA-seq can be guided by transcripts assembled from bulk long-read samples, and demonstrate that noncoding transcripts are expressed at similar levels to coding transcripts but are expressed in fewer cells. This study highlights the impact of sequencing depth on transcript assembly.

**Supplementary Information:**

The online version contains supplementary material available at 10.1186/s12864-022-08717-z.

## Introduction

The genomic era has brought about a deep understanding of genomes [1]. The sequencing of human [1], mouse [2] and multiple other genomes has led to an increased understanding of genome structures [3, 4]. In addition, technological advancements have led to the genome-wide annotation and assessment of functional genomic elements. Of particular importance has been the identification of transcribed units, which have been described in multiple species [5–7], making it possible to investigate transcript functions. However, despite decades of research, there remains considerable ambiguity on the full transcriptome [8–13].

Transcripts can be generally divided into two classes based on protein-coding ability [14–16]. While coding transcripts code for proteins, noncoding transcripts do not code for viable proteins [11, 16]. A class of noncoding transcripts at least 200 nucleotides long, termed long non-coding RNA (lncRNA), has attracted a lot of attention. In addition to coding potential there are other critical differences between the behavior of coding and noncoding transcripts: Coding transcripts tend to have higher expression levels, be longer, less likely to be localized to the nucleus, more stable, less tissue-specific, more evolutionarily conserved and contain fewer transposable element (TE)-derived sequences. Noncoding transcripts are the reverse for all of these properties [15, 17]. This suggests that coding and noncoding transcripts may have different levels of complexity for transcript assembly. Indeed, the overlap between noncoding transcript databases is low [18], and, the majority of novel transcripts have been reported to be noncoding transcripts [12, 17].

The low expression, poor concordance between databases and abundance of TE-derived sequences in noncoding RNAs suggest that noncoding transcript assembly is more complicated. Therefore, it is likely that more reads are required to assemble noncoding transcripts, however the full influence of sequencing depth for short and long-reads has not been previously described. In this study, we retrieved 671 publicly available short-read bulk RNAs-seq datasets from a panel of cell types and tissues with various numbers of reads to investigate the impact of sequencing depth on the assembly of coding and noncoding transcripts. We further focused on 150 human pluripotent stem cell (hPSC) samples for which extensive bulk short-read and matching long-read and single cell-RNA-seq (scRNA-seq) data are available. We report that the relationship between the number of reads and the number of recovered transcripts varied based on cell or tissue type, the type of read considered and the nature and expression levels of the transcripts, with the noncoding transcripts consistently benefiting more from deeper sequencing. Overall, we find that sequencing read depth has a relatively minor impact on the detection of coding transcripts, but a major effect on the recovery of noncoding transcripts.

## Materials and methods

### Data analyzed

The short-read data used in this study were from an in-house collection of publicly available data (Supplementary Table 1). A total of 671 cells and tissue types were used for analysis. This collection included 150 human pluripotent cell samples that were previously analyzed in Babarinde et al. (2021). In addition to the short-read samples, four publicly available long-read samples (ENCFF688QGB, ENCFF272VSN, ENCFF954UFG and ENCFF251CBB) sequenced on the PacBio sequencing platform were also retrieved from the ENCODE project [19]. Single-cell data of c11/S0730 cell line (PRJNA631808) was previously described [17].

### Transcript assembly and coding potential Single-cell RNA-seq

The transcript assembly pipeline employed in this study is similar to the one used in Babarinde et al*.* For short-reads, the reads were first aligned to the hg38 version of the human genome using HISAT2 [20]. Transcripts were assembled from the short-read alignment using StringTie [21, 22]. For long-read data, the fastq files were first converted to fasta format and aligned with Minimap2 [23]. Alignments were sorted with SAMtools [24] and transcripts were assembled using StringTie. Transcripts with no inferred strand or those shorter than 200 nucleotides were discarded [17]. Transcript coding potential for each assembly was measured using FEELnc trained using version 34 of human GENCODE [25]. The FASTQ data from c11/S0730 iPSCs (PRJNA631808) was aligned as described in He et al., 2021, using the top 3000 cells. Expression quantification was estimated using StringTie [22]. The expression matrix of the raw counts was normalized using DESeq2 [26].

### Short-read data simulation and long-read subsampling

Two high-depth hPSC samples with over 200 million reads each (SRR597895 and SRR597912) [27], were used for short-read simulation. First, transcript assembly was done using the two samples. The resulting transcripts were then used to build a reference for RSEM [28]. The counts, model and theta were estimated using *rsem-calculate-expression* with bowtie2 [29] alignment options. Finally, read simulations were generated using *rsem-simulate-reads*. For long-reads, H9 hESC data (PRJNA63104) was subsampled using SAMtools [24]. Independent transcript alignments were then carried out for each of the sub-sampled alignments.

## Results

### Relationship between sequencing depth and transcript assembly across cell types and tissues

**Fig. 1:**
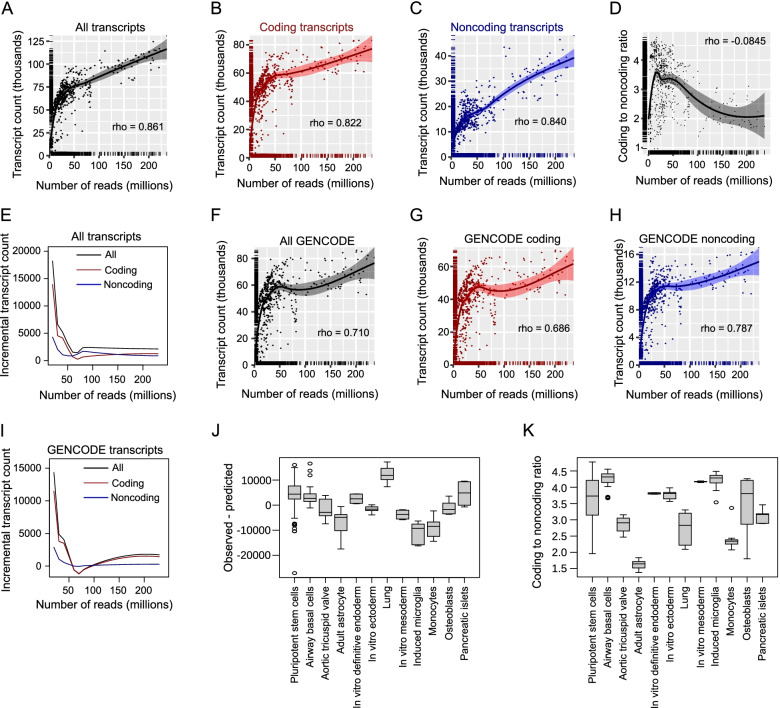
Relationship between the number of reads and assembled transcript count across multiple cells and tissues. Transcript assembly and coding potential evaluation were done for each of the 671 short-read samples from multiple cells and tissues. The relationships between the number of reads and the transcript count for all transcripts (**A**), coding transcripts (**B**) and noncoding transcripts (**C**). For panels **A**- **C** and **F**–**H**, each point represents a sample while the line represents the loess fit with standard error band shown. Rho is the Spearman rank correlation coefficient. The distributions of the samples are shown on the axes. **D**. Number of reads versus coding to noncoding ratio. **E**. Increment in the number of transcripts per additional 10 million reads. The numbers of transcripts from 0 to 240 million reads with 10 million increments were predicted from loess fits in panels **A-C**. The number of transcripts due to additional 10 million read increment in read depth is shown on the vertical axis. Numbers of reads versus the number of transcripts for all GENCODE-annotated transcripts (**F**), GENCODE-annotated coding transcripts (**G**) and GENCODE-annotated noncoding transcripts (**H**). Assembled transcripts that were not found in GENCODE annotation were not included **I**. Increment in the number of transcripts inferred from loess prediction per additional 10 million reads for GENCODE-annotated transcripts. **J**. The difference between the observed and the predicted numbers of transcripts based on the loess prediction of all the 671 samples. Cells or tissues with at least 8 biological or technical replicates in the dataset are presented. **K**. The coding to noncoding ratio varied across cells and tissue types. Only cells or tissues with at least 8 biological or technical replicates are shown

To understand the relationship between sequencing depth and transcript assembly, we assembled transcripts from 671 human samples with sequencing depths ranging from 165,322 to 236,275,714 reads (mean of 38,132,992 and median of 25,717,722 reads) (Table S1). The analyzed samples were all paired-end short-reads of which at least 70% aligned to the hg38 genome. We investigated the read depth at three levels: The first level is the total number of reads. The second level is the number of reads that mapped to the genome. The third level is the number of reads that were assigned to a transcript, representing the number of reads that contribute to transcript assembly. The assembled transcript sets included both GENCODE-annotated and non-GENCODE transcripts. Our analyses showed that the number of transcripts retrieved is proportional to the sequencing depth, although the relationship is not linear (Fig. 1A). Overall, Spearman’s correlation coefficient (rho) was 0.861. The transcript set from the assembly of each sample was then divided into coding (Fig. 1B) and noncoding (Fig. 1C) transcripts based on the FEELnc score [25]. In almost all samples, more coding transcripts were found than noncoding transcripts. The correlation between the number of transcripts and sequencing depth for coding and noncoding transcripts were 0.822 (Fig. 1B) and 0.840 (Fig. 1C), respectively, reflecting a slightly higher correlation for noncoding transcripts. Because the samples were not evenly distributed across the depths, we grouped the samples into 10 million read bins. The boxplots of the binned samples revealed that the positive relationships between the transcript count and read depth still held (Fig. 1SA). The analyses involving the number of mapped reads (Figure S1B) and the number of reads assigned to transcripts (Figure S1C) produced similar correlations. In fact, there was a high correlation between the total number of reads, number of mapped reads and number of reads assigned to transcripts (Spearman rank correlation coefficient for all reads versus mapped reads = 0.996; all reads versus transcript-assigned reads = 0.959; mapped reads versus transcript-assigned reads = 0.971).

The slightly higher correlations between noncoding transcripts and the sequencing depth suggest that the proportion of coding transcripts might be different across sequencing depths. Indeed, samples with relatively shallower depths tended to have higher coding to noncoding ratios (Fig. 1D). This indicates that coding sequence transcripts saturate at relatively shallow sequencing depths. The loess fit shows that the ratio slightly increases initially, and starts dropping at around 50 million reads and was lower than 3 for the highest depth samples. This suggests that deeper sequencing leads to an increased number of noncoding transcripts.

We next checked for the increase in the number of transcripts for every 10 million additional reads using the loess fit. The result shows that the incremental number of transcripts was higher at shallow depths than at high depths (Fig. 1E). The coding transcripts reached the lowest level of increase at 70 million reads, demonstrating that coding transcripts are assembled from a relatively small number of reads. After 100 million reads, the incremental numbers of the transcripts remained relatively similar, possibly caused by the assembly of transcripts specific to each sample. We therefore focused only on GENCODE annotated transcripts. Interestingly, the result remained essentially the same, and noncoding transcripts had a higher correlation between the number of retrieved GENCODE transcripts and the numbers of reads for all GENCODE-annotated transcripts (Fig. 1F), coding transcripts (Fig. 1G) and noncoding transcripts (Fig. 1H). The incremental number of transcripts in the assembly of GENCODE-annotated transcripts with increasing numbers of reads (Fig. 1I) was also similar to what was observed in all transcripts, and most transcripts were assembled with a relatively shallow number of reads. These results show that the relationship between the number of reads and the number of assembled transcripts is not just due to the inclusion of more transcriptional noise, but reflects the ability to retrieve more (potentially) genuine transcripts.

The inclusion of samples from different cell and tissue types assumes that transcript abundance and coding proportion are similar across cell types. However, the number of transcripts can vary across tissues [30]. To explore this, we selected the 12 cells or tissues that had at least eight biological replicates from our dataset. We checked the difference between the actual number of transcripts assembled and the number of transcripts predicted from the read depth using the model in Fig. 1A-C. The results showed that some tissues have positive observed-predicted differences, indicating that the number of transcripts retrieved was more than the number predicted, while others have negative observed-predicted differences, indicating the opposite (Fig. 1J). Interestingly, this pattern was the same for both coding and noncoding transcripts (Figure S1D, E). Intriguingly, there was a rough inverse relationship between the observed-predicted transcript counts between coding and noncoding transcripts (Figure S1D, E). Furthermore, the ratio between coding and noncoding transcripts is heterogenous (Fig. 1K). These results suggest that the relationship between read depth and the number of retrieved transcripts varies across cells or tissues, and that the coding to noncoding ratio varies substantially across different cell types and tissues.

### Impacts of sequencing depth on human pluripotent stem cell transcript assembly

We then analyzed a single cell type in depth. For this, we focused on human pluripotent stem cells (hPSCs). There are widespread problems in metadata annotations of sequencing samples [31–33], hence we used the transcriptome assembly from a set of 150 RNA-seq samples that we previously computationally verified to be normal undifferentiated hPSCs [17]. The assembly from that study comes in two forms, an unfiltered set, containing 272,209 lower confidence transcripts, and a more confident 101,492 filtered transcript set, including 7,261 novel and 19,575 variant transcripts. We defined novel transcripts as transcripts that did not overlap any GENCODE exon, and variant transcripts that were different isoforms of a GENCODE gene [17].

**Fig. 2:**
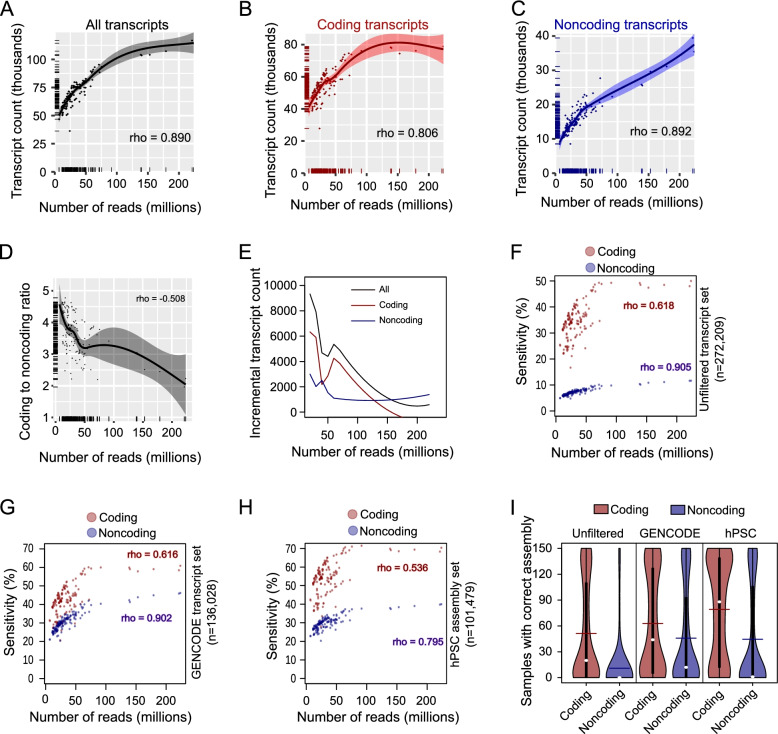
Sequencing depth is positively correlated with transcript count and sensitivity in human pluripotent stem cells. **A-C**. Number of reads versus the number of transcripts assembled from 150 computationally verified hPSC samples. The numbers of all hPSC transcripts (**A**), coding transcripts (**B**) and noncoding transcripts (**C**) are shown on the vertical axes. The lines indicate the loess fits with standard error bands shown. Rho is the Spearman rank correlation coefficient. The distributions of the samples are shown on the axes. **D**. Number of reads versus the coding to noncoding ratio. **E**. Increment in the number of transcripts per additional 10 million reads. The numbers of transcripts are predicted from the loess fits in panels A-C. **F**. Sensitivity versus the number of reads using unfiltered transcript set assembled from the merged alignment of all the 150 hPSCs. **G**. Sensitivity versus the number of reads using only GENCODE-annotated assembled transcript set. The hPSC transcripts that were not found in GENCODE annotation were excluded. **H**. Sensitivity versus the number of reads using hPSC assembly. The hPSC assembly, published in Babarinde et al*.* includes additional transcripts that were not found in GENCODE annotation but excludes GENCODE transcripts that are not expressed in hPSCs. **I**. The number of samples in which the transcripts are assembled. For each category of transcripts, the number of samples for which the transcripts were correctly assembled was computed

We investigated the effect of read depth on the number of transcripts retrieved in hPSCs. We confirmed the positive relationships between the read counts and all transcript count (Figs. 2A, S2A), coding transcript count (Figs. 2B, S2A) and noncoding transcript count (Figs. 2C, S2A). The relationships between transcript count and the number of reads were generally stronger in hPSC samples than in the multi-sample analyses (Fig. 2A-C). Similar results were also found in the relationship between transcript counts and the number of mapped reads or the number of reads assigned to transcripts (Figure S2B, C). Unsurprisingly, there is a high correlation between the number of reads, the number of mapped reads and the number of reads assigned to transcripts (Spearman rank correlation coefficient for all reads versus mapped reads rho = 0.991; all reads versus transcript-assigned reads = 0.958; mapped reads versus transcript-assigned reads = 0.970). Interestingly, there was a significant negative relationship between the coding to noncoding ratio and read number, as deeper sequenced samples tended to have a higher proportion of noncoding transcripts (Fig. 2D). The incremental numbers of transcripts per increasing reads, predicted from the loess fit, was also different between coding and noncoding (Fig. 2E). At around 150 million reads, the incremental number of coding transcripts found was zero, suggesting that sequencing had reached full saturation for coding transcripts. However, for noncoding transcripts, even at 230 million reads (the largest single library in our data set), the incremental number of assembled transcripts did not reach zero, indicating that additional noncoding transcripts continued to be detected from deeper sequencing.

We then set out to explore the sensitivity of the transcript assembly. We defined false positives as a transcript that matched to the unfiltered superset of hPSC transcripts (*n* = 272,209) [17]. We checked the sensitivity of each sample in terms of the percentage of the transcripts that were correctly assembled, relative to the hPSC unfiltered superset. Our result showed that the sensitivity of coding transcripts is consistently higher than that of noncoding transcripts (Fig. 2F). The sensitivity of coding transcripts seemed to reach a peak of about 50%, after 70 million reads. For all the samples, the sensitivity of noncoding transcripts never exceeded 15%. As expected, deeper samples have higher sensitivity. This may be at least partly due to differences between the ratios of coding and noncoding transcripts in the transcript assemblies, as unfiltered transcripts had substantially more noncoding compared to the filtered and GENCODE (Figure S2D). This suggests that the filtering process disproportionately affected noncoding transcripts.

Since the unfiltered transcript set likely contains many unreliable transcripts, we repeated the hPSC transcript assembly sensitivity analysis by focusing only on the unfiltered hPSC transcripts that are in GENCODE (Fig. 2G). The patterns were essentially the same: Noncoding transcripts tended to have lower overall sensitivity, although the overall sensitivity is improved. Previous studies have reported that the GENCODE annotation is not definitive [11, 17, 34, 35]. Therefore, we repeated the analyses using the hPSC filtered transcript set which included transcripts with detectable expression in at least 50 hPSC samples [17]. The results remain essentially the same, except that the sensitivity of coding transcripts goes up (Fig. 2H). One of the steps in filtering the hPSC transcripts is based on the number of samples in which a transcript is expressed [17], hence we checked the number of expressing samples and found that coding transcripts tended to be more broadly expressed (Fig. 2I). For coding and noncoding transcripts, transcripts that are correctly assembled in more samples (Figure S2E) or detected in more samples (Figure S2F), tend to have higher expression levels. Finally, we decided to focus on transcripts that were correctly assembled in multiple samples. These transcripts are less likely to be transcriptional noise as it is unlikely for the transcriptional noise to have the same splicing patterns across multiple samples. We therefore made assemblies of hPSC transcripts with the same assembly in at least 2, 5, 10, 20 and 50 samples. The number of assembled transcripts reduced with the number of samples in which the transcripts were assembled. Importantly, the Spearman’s rank correlation coefficients were consistently higher for noncoding transcripts than coding transcripts (Table S1). Taken together, these results show that whilst coding transcripts saturate rapidly at relatively low numbers of reads, the number of noncoding transcripts continues to increase with sequencing depth.

We next checked if noncoding transcript assembly would reach a saturation at much deeper sequencing depth. However, the deepest hPSC sample in our dataset (SRR597895) had less than 240 million reads. Therefore, we checked the distribution of coding and noncoding transcripts in the unfiltered superset of hPSC transcripts obtained from the merging of all the 150 hPSC samples (with > 5.5 billion paired reads) and found that 47.14% were coding while 52.86% were noncoding. This suggests that noncoding transcripts would continue to benefit from higher depth but the benefit slowed down. We therefore merged the top two, four and six high-depth hPSC samples and assembled the transcripts from the merged samples. The results show that the numbers of coding transcripts were similar, but the noncoding transcripts continue to benefit more (Figure S2G). However, the difference in the number of noncoding transcripts between the top 4 and top 6 assemblies suggest that noncoding transcripts benefit less from higher depths. It is important to note that combining multiple samples might introduce bias in the assembly. To mitigate this, we therefore subsampled 500, 1,000, 1,500 and 2,000 paired reads from the merged alignments of 150 hPSC samples. Interestingly, the increase in the number of assembled transcripts for both coding and noncoding transcripts across read depths were similar at much higher depths (Figure S2H). These results suggest that advantage that noncoding transcript assembly have over coding transcripts eventually diminishes at extremely high sequencing depths.

### Effects of read depth and biological replicates on transcript assembly

**Fig. 3:**
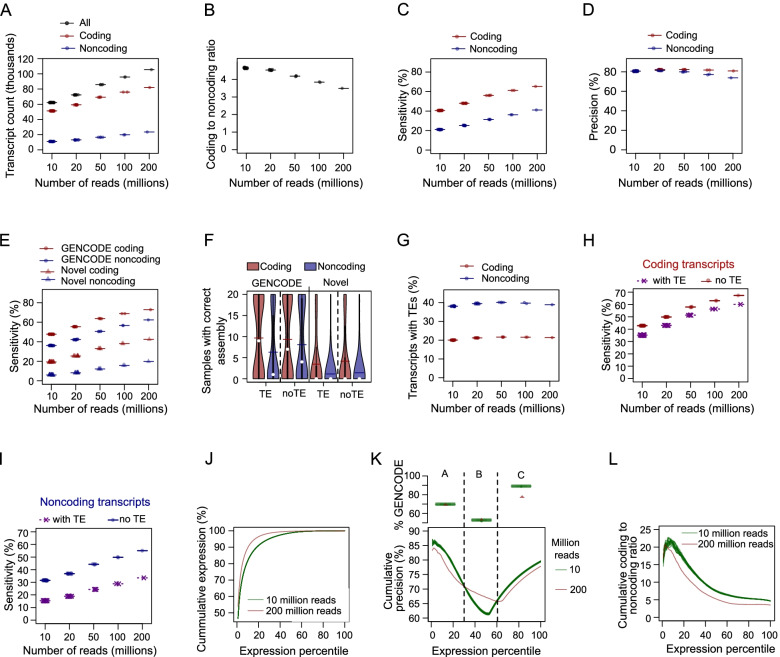
Effect of sequencing depth on the numbers and correctness of transcripts assembled from simulated short-read data**.** 10 million (*n* = 20), 20 million (*n* = 10), 50 million (*n* = 4), 100 million (*n* = 2) and 200 million (*n* = 1) hPSC reads were simulated using SRR597912 and SRR597895 reads. Transcript assembly and coding potential evaluation were done for each simulated read sample. The relationship between the number of reads and transcript counts (panel** A**), coding to noncoding ratio (panel** B**), sensitivity (panel** C**) and precision (panel** D**) are presented for each sample. The transcript assembly obtained from the merged SRR597912 and SRR597895 reads was used as the reference for the estimation of sensitivity and precision. **E**. Sensitivity is positively correlated with the number of reads for GENCODE-annotated and novel transcripts. Samples with the same read numbers produced similar numbers of transcripts. **F**. The numbers of samples for which different categories of transcripts were correctly assembled. The assemblies of 20 replicates of 10 million reads were investigated to highlight correctly assembled transcripts. **G**. The proportion of TE-containing transcripts was similar across various read depths. The sensitivity of assemblies of coding transcripts (**H**) and noncoding transcripts (**I**) showing the differences between TE-containing and TE-lacking transcripts. **J**. Cumulative expression of assembled transcripts. The transcripts were ranked based on expression level, and the cumulative expression is presented in percentages. **K**. Cumulative precision of transcripts based on the expression levels. Based on the expression levels and the cumulative precision in 200 and 10 million read assemblies (lower panel), the transcripts were grouped into three classes. The upper panel shows the percentages of GNCODE-annotated transcripts in the three classes of transcripts. **L**. Cumulative coding to noncoding ratio varied with expression levels and the numbers of reads

The results in Fig. 2A-H involved hPSC samples from different sources. The results might therefore reflect both the effects from the number of reads and factors independent of sequencing depth. To investigate the impact of the number of reads on transcript assembly from a homogenous background, we assembled transcripts from different numbers of simulated reads. Individual transcript assembly confirmed that the number of transcripts increases with the number of reads, and the fraction of coding transcripts tended to dominate the total number of transcripts (Fig. 3A). Similarly, the coding to noncoding ratio tended to decrease with sequencing depth, confirming that deeper sequenced samples can indeed retrieve more noncoding transcripts (Fig. 3B). The sensitivity of the assembly was also increased with sequencing depth, with the sensitivity in coding transcripts consistently higher than the sensitivity in noncoding transcripts (Fig. 3C). Interestingly, the precision of the coding and noncoding transcripts for lower depths were similar (Fig. 3D), suggesting that the increase in sensitivity is not always accompanied by a decrease in precision.

To investigate the impact at much higher depths, we simulated up to 1 billion paired short reads. The result shows that up to 1 billion reads, the number of assembled coding transcripts was higher than the number of assembled noncoding transcripts (Figure S3A). Importantly, the result shows that the number of assembled noncoding transcripts tended to flatten after 600 million reads. Consistent with the transcript counts, the coding to noncoding ratio continues to drop, but the difference becomes smaller at around 600 million reads (Figure S3B). The analyses of sensitivity further reveal that support for noncoding assembly flattens after 600 million reads (Figure S3C). While the precision of coding transcripts remains more or less similar up to 1 billion read depths, the precision of noncoding transcript assembly decreases with decreasing depth, suggesting that the probability of assembling false noncoding transcripts increases with increasing read depth. These results suggest that noncoding transcript assembly benefits from up to 600 million reads, however after that point the assembly of additional noncoding transcripts becomes less reliable.

The ‘correctness’ of a transcript assembly can also be assessed in terms of transcript completeness at the exon or splice level [17]. Transcript completeness was compared between assemblies from 10 million reads (low depth) and assembly from 200 million reads (high depth). Table 1 shows that the exons of each assembled transcript were mostly either found (100% exon completeness) or were completely missing (0% exon completeness), reflecting the integrity of the assembly. Partial exon overlaps were (surprisingly) not common. Consistent with the results in Fig. 3C, the 200 million deep read assembly was more complete, and coding transcripts had higher exon completeness than noncoding transcripts. A valid transcript should have completely spliced introns, and when we computed splice completeness, we found a similar pattern to exon completeness (Table 1). These results highlight the importance of read depth and the difficulty in assembling noncoding transcripts.

**Table 1:** Exon and splice completeness of transcript assemblies

Read type	Feature	Read count^a^	Number	Coding transcripts (%)^b^		Noncoding transcripts (%)^b^	
				**Complete (100%)**	**Missing (0%)**	**Complete (100%)**	**Missing (0%)**
Simulated short read	Exon	10 m	20	39.94 (0.1)	56.7 (0.09)	21.01 (0.06)	77.68 (0.07)
	Exon	20 m	10	47.04 (0.07)	50.0 (0.08)	25.14 (0.15)	73.21 (0.15)
	Exon	50 m	4	54.9 (0.11)	42.43 (0.12)	31.22 (0.1)	66.62 (0.1)
	Exon	100 m	2	59.85 (0.03)	37.63 (0.01)	35.98 (0.02)	61.37 (0.05)
	Exon	200 m	1	63.86 (0.0)	33.8 (0.0)	40.82 (0.0)	55.98 (0.0)
	Splice	10 m	20	39.94 (0.1)	56.73 (0.09)	21.01 (0.06)	77.79 (0.08)
	Splice	20 m	10	47.04 (0.07)	50.03 (0.08)	25.14 (0.16)	73.37 (0.16)
	Splice	50 m	4	54.9 (0.11)	42.47 (0.12)	31.22 (0.1)	66.86 (0.1)
	Splice	100 m	2	59.85 (0.03)	37.65 (0.01)	35.98 (0.02)	61.65 (0.07)
	Splice	200 m	1	63.85 (0.0)	33.83 (0.0)	40.82 (0.0)	56.37 (0.0)
Long read	Exon	25%	4	61.15 (0.15)	34.66 (0.17)	39.27 (0.13)	53.62 (0.18)
	Exon	50%	2	76.28 (0.06)	20.0 (0.05)	58.78 (0.02)	33.27 (0.05)
	Exon	75%	1	87.54 (0.0)	10.05 (0.0)	77.33 (0.0)	17.01 (0.0)
	Splice	25%	4	61.0 (0.14)	34.68 (0.17)	39.35 (0.15)	53.79 (0.16)
	Splice	50%	2	76.12 (0.04)	20.02 (0.05)	58.87 (0.04)	33.37 (0.08)
	Splice	75%	1	87.35 (0.0)	10.06 (0.0)	77.34 (0.0)	17.08 (0.0)

Splice or exon completeness was estimated as the percentage of the splice or exon of the reference transcript that is correctly assembled in the new assembly. Transcripts with partial splice or exon assembly are not shown in the table. For multiple samples, the average percentages were presented, with the standard deviations in parentheses

^a^For simulated short reads, read counts were in million paired read whereas for long read samples, read counts were in the percentage of total alignment

^b^Completeness was given as the average of the percentages of reference transcripts. The values in parentheses are the standard deviations computed from multiple samples

The reference transcripts used for the simulation comprised the GENCODE annotated transcripts and the novel or variant transcripts defined in Babarinde et al. (2021). The novel transcripts contained a higher percentage of noncoding transcripts (Figure S3D). Across both the GENCODE and novel transcript sets, coding transcripts tended to have higher expression levels (Figure S3E). Interestingly, the expression levels of novel transcripts tended to be higher than that of GENCODE annotated transcripts. The results further highlight the fact that noncoding transcripts are more difficult to assemble and are depleted in GENCODE annotations.

We next checked if the sensitivity of the transcript assembly is similar between novel and GENCODE transcripts. Across different read depths, the GENCODE annotated set consistently had higher sensitivity (Fig. 3E), suggesting that the novel transcripts are more difficult to assemble. One reason may be because of the higher proportion of transposable element (TE) fragment sequences in novel transcripts (Figure S3F). Specifically, the GENCODE coding transcript set with the highest assembly sensitivity had the lowest TE content while the novel noncoding transcript set with the lowest assembly sensitivity contained the highest proportion of TE-containing transcripts. We next checked how the number of expressing samples is affected by TE presence and coding ability in GENCODE and novel transcript sets (Fig. 3F). This analysis was done using 20 replicates of 10 million reads. GENCODE annotated transcripts tended to be more broadly expressed than novel transcripts, and coding transcripts tended to have broader expression than noncoding transcripts (Fig. 3F). Also, transcripts with no TEs tended to be more broadly expressed, especially in GENCODE annotated noncoding transcripts. Interestingly, increases in sequencing depth do not seem to substantially improve the proportion of TE-containing transcripts in the assembly (Fig. 3G). However, the sensitivity of the assembly increases with depth for TE-containing and TE-lacking transcripts in both coding (Fig. 3H) and noncoding transcripts (Fig. 3I). Interestingly, the difference in sensitivity between TE-containing and TE-lacking transcript assembly tends to be higher in noncoding than in coding transcripts (Fig. 3H, I). These results suggest a complex impact of TE presence in transcript assembly from short-read data**.**

Next, we investigated the impact of sequencing depth on transcript steady-state (expression) levels. First, we investigated the cumulative RNA levels in shallow-depth samples (10 million reads) and a high-depth sample (200 million reads). The assembly from 200 million reads reached saturation more quickly than the assemblies of 10 million reads (Fig. 3J). For example, 95% of all detected RNAs from the high depth sample were found in the top 17% of highly expressed transcripts. On the contrary, the top 30% of the expressed transcripts in the shallow samples contributed 95% of all expressed RNAs. The investigation of transcript precision showed that transcripts can be classified into three groups based on the expression-ranked assembly precisions of shallow-depth (10 million read) and high-depth (200 million read) samples (Fig. 3K). Groups A (top 30 percentile) and C (lowest 40 percentile) transcripts have higher precisions in the shallow-depth samples while group B (30–60 percentile) transcripts have higher precision in the high-depth sample. Group B transcripts have the lowest percentage of GENCODE transcripts suggesting that a substantial proportion of the novel transcripts have intermediate expression levels. Interestingly, there are no major differences in the percentages of GENCODE transcripts in groups A and B across low-depth and high-depth samples. However, group C transcripts have a higher GENCODE percentage in low-depth than high-depth samples. Coding to noncoding ratio falls very rapidly with expression (Fig. 3L), highlighting that the expression level considered can affect the ratio observed. For example, if the top 10% of transcripts were considered, the set would have a similarly high coding to noncoding ratio in 200 million reads as in the 10 million read assemblies. These results demonstrate the effect of read depth on transcript expression level.

### Effect of sequencing depths on transcript assembly in long-read data

**Fig. 4:**
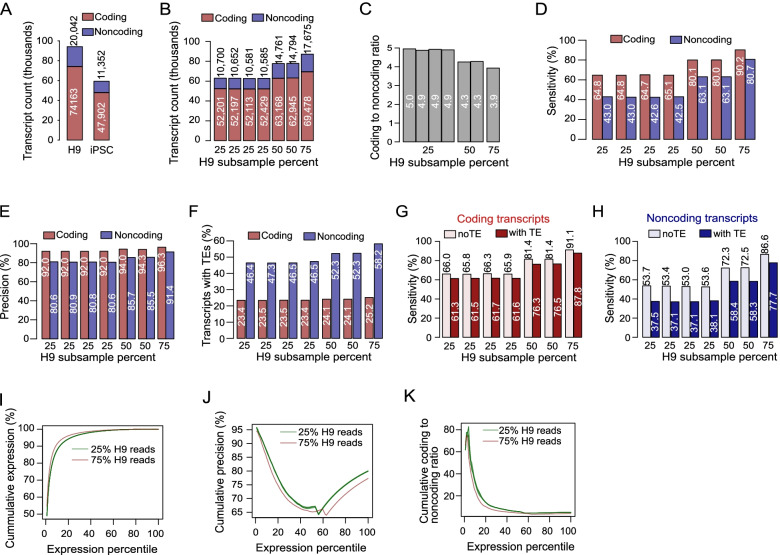
Effect of sequencing depth on the numbers and correctness of transcripts assembled from long-reads data.** A**. Transcript counts from iPSC and H9 human cell lines. 25% (*n* = 4), 50% (*n* = 2) and 75% of the H9 reads were independently sampled without replacements. **B**. Numbers of assembled transcripts from down-sampled H9 long-read data. Effects of down-sampling on coding to noncoding ratio (panel **C**), sensitivity (panel** D**) and precision (panel **E**). The transcript assembly from all the H9 long-read data was used as the reference for the computations of sensitivity and precision. **F**. The proportions of TE-containing transcripts across various depths. The sensitivity of the assemblies with or without TEs in coding (**G**) and noncoding (**H**) transcript sets. **I**. The cumulative expression of transcripts ranked by expression levels in H9 samples for 25% and 75% subsampled read assemblies. **J**. The cumulative precision of the expression-ranked transcripts for 25% and 75% subsampled read assemblies. **K**. The cumulative coding to noncoding ratio of the expression-ranked transcripts

Recent studies have demonstrated the advantages of long-read sequences over short-read sequences for genome and transcript assembly [36–39]. We therefore investigated the effect of sequencing depths on long-read sequences from the PacBio platform. We retrieve the long-read data from H9 (~ 25 million reads) cells and iPSCs (~ 7 million reads) and assembled 94,205 (74,163 coding) transcripts from H9 and 59,254 transcripts (47,902 coding) transcripts from iPSCs (Fig. 4A). These corresponded to a 3.7 and 4.2 coding to noncoding ratio in H9 and iPSCs, respectively.

Because of the limited number of iPSC-sequenced long-read samples, we focused on only the deeper sequenced H9 cell line for the subsequent analyses. To assess the impact of long-read depth, H9 reads were randomly subsampled to 25% (four replicates), 50% (two replicates) and 75% (one replicate). Assemblies with a similar number of reads had similar numbers of transcripts (Fig. 4B). We confirmed that the number of reads correlated with the number of assembled transcripts, and the coding to noncoding ratio is higher in samples with fewer reads (Fig. 4B, C). For sensitivity and precision, the transcript set assembled from the full H9 dataset reads was used as a reference. While the sensitivity was noticeably correlated with the number of reads (Fig. 4D), the precision was not substantially affected (Fig. 4E). This suggests that increasing read depth led to the assembly of more reliable transcripts. Additionally, comparison of the transcripts relative to the reference also shows two clear peaks in the exon and splice completeness (Table 1). The results show that read depth affects the composition and integrity of the assembled transcripts.

To investigate how long read data perform with respect to TE presence, we checked the proportion of TE-containing transcripts across the read depths. While the proportion of TE-containing transcripts remain fairly stable for coding transcripts, the proportion of TE-containing noncoding transcripts tend to increase across depths (Fig. 4F). Interestingly, the assembly sensitivity increases for both TE-containing and TE-lacking transcripts in both coding (Fig. 3G) and noncoding (Fig. 3H) sets. However, the difference in the sensitivity of TE-containing and TE-lacking assembly was higher in noncoding that coding transcript sets. The results highlight the advantage of long-read data in assembling TE-containing transcripts, which are predominantly noncoding.

We also investigated the impact of transcript expression level on transcript assembly from long-read data. We ranked the transcripts by expression in the 25% and 75% samples. The analyses of cumulative expression show that the top 10% of the transcripts account for about 90% of RNAs in the 25% subsamples, and about 93% of the total RNAs in 75% subsamples (Fig. 4I). The precision of transcript assembly also varied by expression level (Fig. 4J). Mainly, the overall precision tended to be higher in the 25% samples, suggesting that fewer novel transcripts are retrieved from 25% samples. The coding to noncoding ratio varied greatly across the expression profiles (Fig. 4K). The top 5% of the transcripts had a ratio of ~ 80 while the ratio fell to < 10 when the top 40% of the transcripts are considered. These results reflect the effect of read depths and the composition of the transcripts greatly depends upon the expression thresholds set.

### Heterogeneity of the assembled transcripts at single cell levels

Unlike bulk short and long-read samples, single cell RNA-seq (scRNA-seq) read coverage is typically too low for transcript assembly. In scRNA-seq reads tend to be heavily 3’ biased, the data is sparse and suffers from transcript dropout from individual cells, and analysis is generally gene-wise, rather than transcript-specific [40]. However, transcript assemblies from matching bulk samples can help guide transcript analysis [41]. We reanalyzed the single cell RNA-seq (scRNA-seq) data from the c11 iPSC cell line [17]. The alignments were split into barcodes representing cells from which the RNAs were sequenced. First, we used the short-read assembly derived from the two largest short-read samples, SRR597912 and SRR597895 [27]. We checked the relationship between the barcode tag count and the number of transcripts with detectable expression in each cell. We found that the total sequence tag count was positively correlated with the number of coding (Figure S4A) and noncoding (Figure S4B) transcripts with detectable expression. Cells with more reads tended to have a larger number of detectable transcripts. As expected, the number of coding transcripts with detectable expression was more than the number of noncoding transcripts with detectable expression (Figure S4A, B). This suggests that the number of reads strongly affects single cell transcript quantification.

**Fig. 5:**
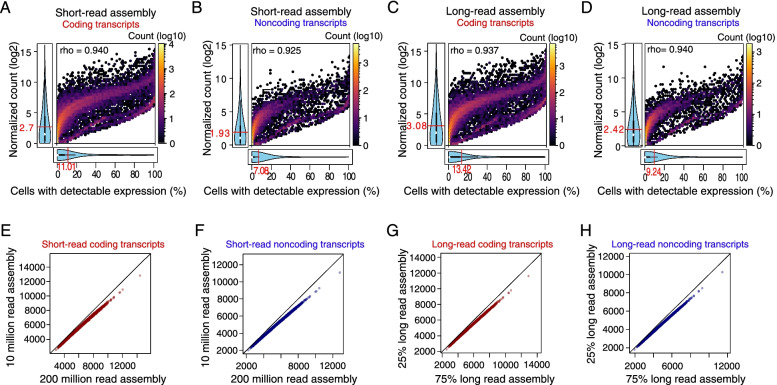
Single cell data reveal the heterogeneity of transcripts assembled from different data sources and read depths. The top 3,000 barcodes, corresponding to 3,000 cells were extracted from the alignment of c11/S0730 cell line. Panels **A-B** and **E–F** show the results from short-read transcript assembly using SRR597912 and SRR597895, and panels** C-D- **and** G-H** show the results for the transcript assembly from long-read H9 sample. The normalized count of the coding (panel** A**) and noncoding (panel** B**) transcripts are positively correlated to the percentages of cells in which the transcript expressions are detected in scRNA-seq data. The normalized counts of the coding (panel** C**) and noncoding (panel** D**) transcripts from long-read assembly are positively correlated to the percentages of cells with detectable expression in scRNA-seq data. The means of the expression levels and percentages of cells with detectable expressions are shown in red fonts in panels **A-D**. The numbers of detectable transcripts in scRNA-seq data were compared for transcripts assembled from different read numbers of short-read (panels** E** and **F**) and long-read (panels** G** and **H**) data. For coding (panel** E**) and noncoding (panel** F**) transcripts, more transcripts assembled from 200 million reads were detected in scRNA-seq data than the transcripts assembled from 10 million reads. More coding (panel** G**) and noncoding (panel **H**) transcripts detectable from scRNA-seq data were found for the assembly from 75% long-read data than for the assembly from 25% long-read data. For panels** E–H**, each point represents a cell. The diagonals represent the points of equal numbers of transcripts from the compared assemblies

In short-read bulk samples, we established that coding transcripts tend to be expressed in more samples (Figs. 2I and 3F) and at higher levels (Figure S3D) than noncoding transcripts. We therefore asked if there is a relationship between the expression levels and the percentage of cells with detectable expression. As expected, transcripts with higher expression levels tended to be expressed in more cells for coding (Figure S4C) and noncoding (Figure S4D) transcripts. Using the short-read transcript set with detectable expression in scRNA-seq data, we also found that the expression level and the percentage of cells with detectable expression for the transcripts were higher in coding than in noncoding transcripts (Fig. 5A and B). Short-read transcript assembly likely contains more transcriptional noise than long-read transcript assembly [17]. We therefore repeated the same analyses using the H9 long-read transcript assembly.

We confirmed the positive correlations between the barcode tag counts and the numbers of transcripts with detectable expression for both coding (Figure S4E) and noncoding (Figure S4F) transcript sets. We also confirmed positive correlations between expression levels and the percentage of cells with detectable expression for both coding (Figure S4G) and noncoding transcripts (Figure S4H). However, for transcripts with detectable scRNA-Seq expression, the expression levels of long-read assembled transcripts tended to be higher than the expression levels of short-read assembled transcripts (3.08 versus 2.7 for coding; 2.42 versus 1.93 for noncoding transcripts). Similarly, the percentages of cells with detectable expression when using the long-read transcript assembly (Fig. 5C and D) were higher than the percentages for short-read transcript assembly (Fig. 5A and B). Further, the coefficient of variations of the expression levels were significantly higher for coding than noncoding transcripts in both short-read and long-read assemblies (Figure S4I and J), reflecting higher expression heterogeneity in noncoding transcripts. These results show that a fewer number of expressing cells contribute more to the low expression levels of noncoding transcripts observed in bulk RNA-seq samples.

Finally, we investigated the effect of sequencing depth of the assembly on the number of transcripts with detectable expression in scRNA-seq data. For the short-read transcript assembly, we compared the transcript assemblies from 10 and 200 million reads. In both coding (Fig. 5E) and noncoding (Fig. 5F) transcript sets, the numbers of transcripts with detectable scRNA-seq expressions were higher in 200 million read assembly than in 10 million read assembly. We repeated the same analysis using shallow-depth (25%) and high-depth (75%) long-read data assemblies. The results show that the number of coding (Fig. 5G) and noncoding (Fig. 5H) transcripts from long-read assemblies with detectable scRNA-seq expressions were higher in high-depth assembly than in shallow-depth assembly. These results suggest that scRNA-seq analysis guided by long-read assembly data is more reliable than short-read-guided analysis.

## Discussion

Previous studies have identified differences between coding and noncoding transcripts [16, 17, 42, 43]. Novel annotated transcripts tend to be dominated by noncoding transcripts [11, 16, 17, 44], and even for coding transcripts that have been relatively well-annotated, the precise number of genes remains controversial [10–12, 45, 46]. In this study, we systematically investigated the effect of sequencing depth on transcript assembly for the human genome and particularly for hPSCs. Using short-read data from 671 tissues and cell types, we found that the number of reads is positively correlated with the number of transcripts assembled. The correlation between the sequencing depth and the number of assembled transcripts was higher in noncoding transcripts. Additionally, we have established that the proportion of coding to noncoding transcripts decreases with increases in sequencing depth, indicating that noncoding transcripts benefit from deeper sequencing. However, after 600 million reads, the advantage of deeper sequencing in noncoding assembly tended to disappear as the sensitivity flattens and precision continues to drop. All the data showed a positive correlation between sensitivity and sequencing depth, supporting the benefits of increased sequencing depth for improved transcript assembly.

Several factors affect the number and accuracy of assembled transcripts. As stated above, noncoding transcripts tend to benefit more from higher sequencing depths. A possible reason for the difficulty in assembling noncoding transcripts may be partially due to the presence of TEs or lower apparent expression levels in bulk RNA-seq samples [15, 17, 36]. Highly expressed transcripts tend to be easily assembled, medium levels are more challenging to assemble, whilst low-expressed transcripts are assembled only if they have the support of reference transcripts. We also assessed the impact of long-read sequencing for transcript assembly, and comparing the results from the short-read and long-read assemblies, we demonstrated that long-read sequencing particularly benefits the assembly of TE-sequence containing noncoding transcripts. In conclusion, this study highlights the effects of the sequencing depth on transcript assembly, and how these impact transcript discovery and quantitation.

## Supplementary Information

**Additional file 1.**

**Additional file 2.**

## Data Availability

The data used in this study was from previous studies and was downloaded from the NCBI Sequencing Read Archive, of the Gene Expression Omnibus, or European Read Archive. The accessions used in the manuscript are: SRR597895, SRR597912, PRJNA63104, PRJNA631808 and the accessions listed in Table S1.
