# Supplementary material for: The effects of sequencing depth on the assembly of coding and noncoding transcripts in the human genome
Source: BMC Genomics. 2022 Jul 4;23:487. doi: 10.1186/s12864-022-08717-z (PMC9251931; doi:10.1186/s12864-022-08717-z)
Supplement: Supplementary file 1 — Additional file 1. [file 12864_2022_8717_MOESM1_ESM.pdf]

The effects of sequencing depth on the assembly of coding and noncoding transcripts in the human genome

Isaac Adeyemi Babarinde[1] and Andrew Paul Hutchins[1]

[1]Shenzhen Key Laboratory of Gene Regulation and Systems Biology, Department of Biology, School of Life Sciences, Southern University of Science and Technology, Shenzhen, 518055, China.

Correspondence: babarindeia@sustech.edu.cn; andrewh@sustech.edu.cn

Supplementary Methods

Computation of transcript assembly integrity

We measured the integrity of transcript assembly with two parameters. Sensitivity measures the percentage of reference transcripts captured in the assembly. Precision measures the percentage of the assembled transcripts that are in the reference transcript set. Given the reference transcript set with N transcripts and a sample transcript assembly set with n transcripts, out of which e transcripts are correctly assembled as they are in the reference, sensitivity and precision are computed as follow.

$$Sensitivity = \frac{e}{N} \times 100$$

$$Precision = \frac{e}{n} \times 100$$

Further, transcript completeness was estimated for each transcript at the exon and splice levels. For a reference transcript, exon completeness was computed as the percentage of exons that are found in the closest assembled transcript. A transcript with 100% exon completeness has all the exons retrieved in the assembly. On the other hand, no exon of the transcript with 0% exon completeness was retrieved in the new assembly. Because exon overlap does not always mean

splice overlap, we also computed splice completeness to highlight correct splice junctions in the assembly.

Minimap2 command used for long-read transcript assembly

minimap2 -t 30 -ax splice -uf --secondary=no -C5 -O6,24 -B4 Homo_sapiens.GRCh38.dna.primary_assembly.mmi $fasta > $out.sam 2> $out.err

Alignment subsampling

SAMtools was used for subsampling. For hPSC short-read subsampling, the number of properly paired alignments was first estimated from the merged alignments of the 150 hPSC samples using samtools *flagstats*. The proportion of the alignment that would give 500, 1000, 1,500 and 2,000 properly paired reads was then computed and supplied as option *-s* in *samtools view.* Transcript assembly was then done using the subsampled alignments.

Tables and their legends

Table S1: Table summarizing the 671 short-read bulk paired-end RNA-seq samples. Columns include the sample description (the cell type or tissue as described in the study), the SRA or ERA accession number, and the total (unmapped read count).

Table S2: Spearman's rank correlations for hPSC transcripts correctly assembled from multiple samples. The correlations between the sequencing depth and the number of transcripts correctly assembled in at least 2, 5, 10, 20 or 50 samples were computed.

Minimum sample count	All	Coding	Noncoding
2	0.7570	0.6922	0.9257
5	0.7422	0.6787	0.9226
10	0.7282	0.6633	0.9171
20	0.7043	0.6390	0.9082
50	0.6349	0.5625	0.8702

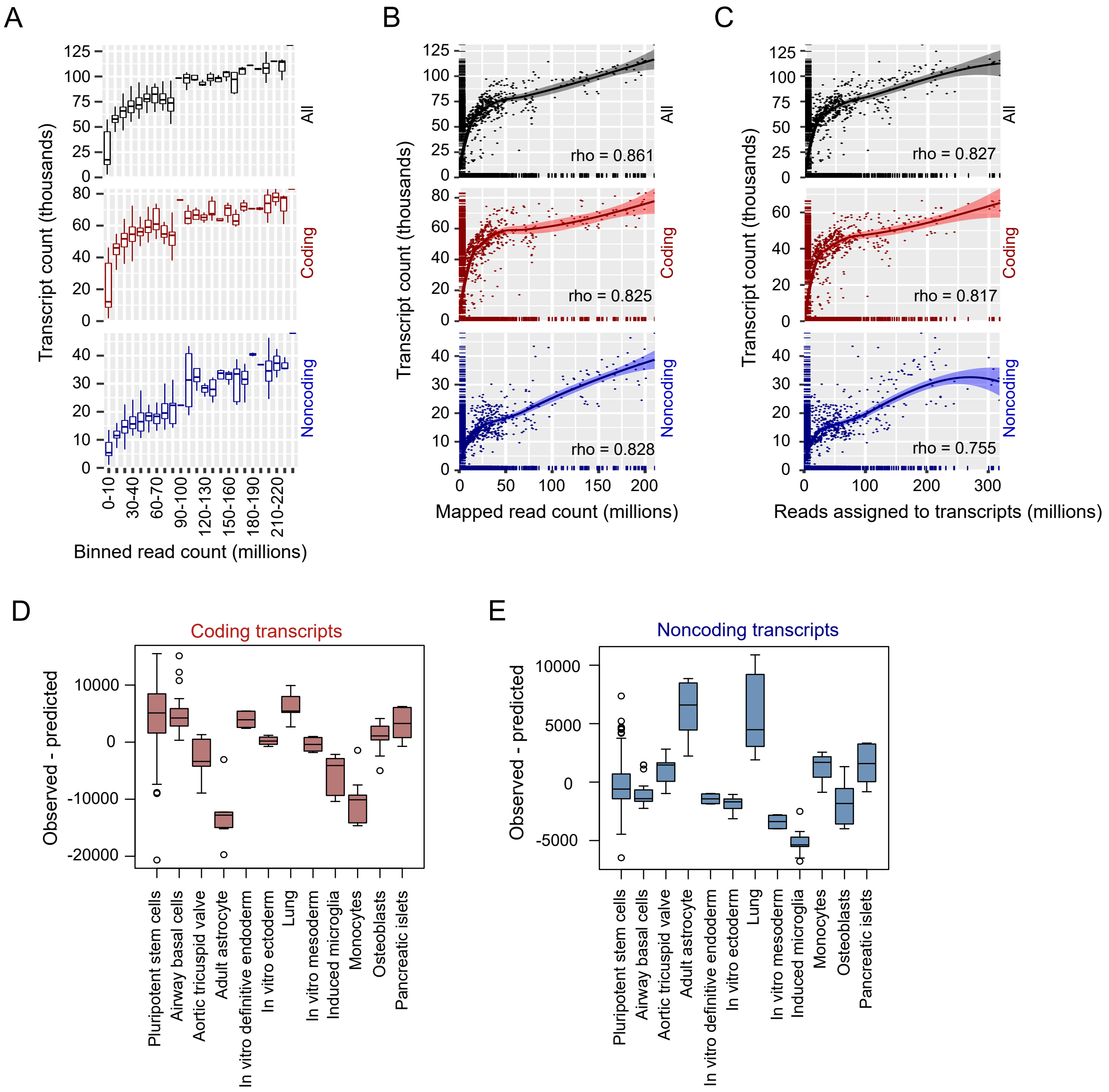

A
B
C
Transcript count (thousands)
All
Coding
Noncoding
0-10
30-40
60-70
90-100
120-130
150-160
180-190
210-220
Binned read count (millions)
rho = 0.861
rho = 0.825
rho = 0.828
Mapped read count (millions)
rho = 0.827
rho = 0.817
rho = 0.755
Reads assigned to transcripts (millions)
D
Coding transcripts
Observed - predicted
Pluripotent stem cells
Airway basal cells
Aortic tricuspid valve
Adult astrocyte
In vitro definitive endoderm
In vitro ectoderm
Lung
In vitro mesoderm
Induced microglia
Monocytes
Osteoblasts
Pancreatic islets
E
Noncoding transcripts

Figure S1

Figure S1: Impacts of different counts of read depths on transcript assembly in multiple cells and tissues. For each sample, the number of aligned reads and the number of reads assigned to transcripts were computed. **A**. Boxplots showing the numbers of transcripts retrieved from binned read counts. The samples were grouped into bins of 10 million reads. **B**. Relationship between the transcript count and the number of mapped reads. **C**. Relationship between the transcript count and the number of reads assigned to transcripts. For figures **S1A-C**, all transcripts are shown in the upper panels, coding transcripts are shown in the middle panels while noncoding transcripts are shown in the lower panels. Rho is the Spearman rank correlation coefficient. Sample distributions are shown on the axes, while the bands represent the standard error of the loess predictions in figures **1B-C**. The difference between the observed and the expected numbers of coding (**panel D**) and noncoding (**panel E**) transcripts based on the loess prediction of all the 671 samples. Cells or tissues with at least 8 biological or technical replicates in the dataset are shown.

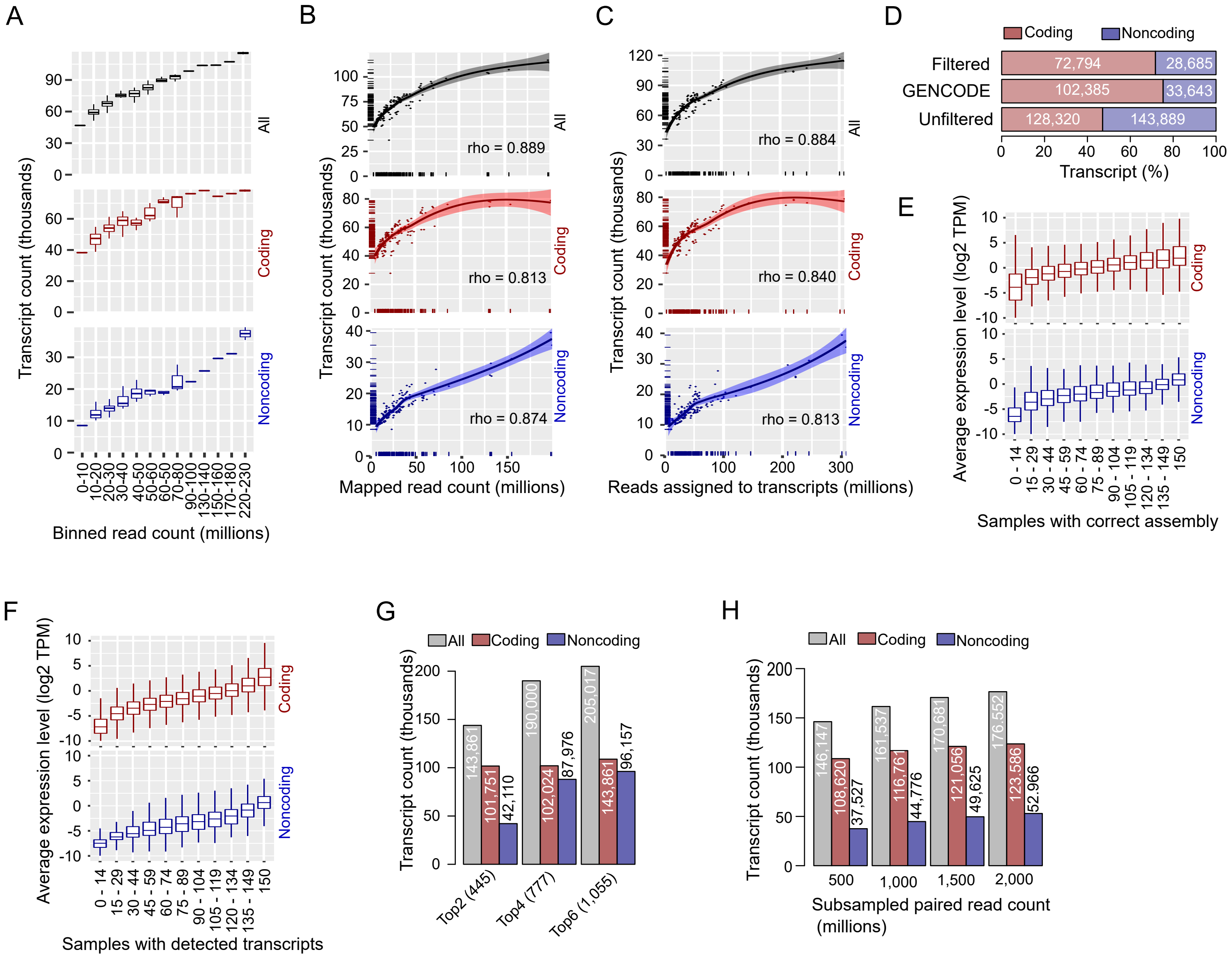

A
B
C
D
E
F
G
H
Transcript count (thousands)
All
Coding
Noncoding
0-10
10-20
20-30
30-40
40-50
50-60
60-50
70-80
90-100
130-140
150-160
170-180
220-230
Binned read count (millions)
rho = 0.889
rho = 0.813
rho = 0.874
Mapped read count (millions)
rho = 0.884
rho = 0.840
rho = 0.813
Reads assigned to transcripts (millions)
Filtered
GENCODE
Unfiltered
72,794
28,685
102,385
33,643
128,320
143,889
Transcript (%)
Average expression level (log2 TPM)
0 - 14
15 - 29
30 - 44
45 - 59
60 - 74
75 - 89
90 - 104
105 - 119
120 - 134
135 - 149
150
Samples with correct assembly
Samples with detected transcripts
143,861
101,751
42,110
190,000
102,024
87,976
205,017
143,861
96,157
Top2 (445)
Top4 (777)
Top6 (1,055)
146,147
108,620
37,527
161,537
116,761
44,776
170,681
121,056
49,625
176,552
123,586
52.966
500
1,000
1,500
2,000
Subsampled paired read count
(millions)

Figure S2

Figure S2: Impacts of different counts of read depth on transcript assembly from short-read bulk RNA samples of human pluripotent stem cells. A. Boxplots showing the numbers of hPSC transcripts retrieved from binned read counts. The samples were grouped into bins of 10 million reads. **B**. Transcript count is positively correlated with the number of mapped reads**. C**. Transcript count is positively correlated with the number of transcript-overlapping reads. The transcript-overlapping reads are more likely to have contributed to the transcript assembly**. D.** The distribution of coding and noncoding transcripts in the unfiltered, GENCODE and filtered hPSC transcript assemblies. The filtered assembly is the hPSC assembly (Babarinde *et al.*, 2021). **E**. Relationship between the transcript expression levels and the number of samples in which the transcripts are correctly assembled. The coding transcripts are shown in the upper panel while the noncoding transcripts are shown in the lower panel. **F.** Relationship between the transcript expression levels and the number of samples with detectable expression. G The numbers of transcripts assembled from the top high-depth hPSC samples. The total numbers of reads are shown in parentheses for top2 (SRR597895 and SRR597912), top4 (SRR597895, SRR597912, SRR597894 and SRR597893) and top6 (SRR597895, SRR597912, SRR597894, SRR597893, SRR574820 and SRR574821) samples. **G.** The number of transcripts assembled from subsampled hPSC alignments. Different numbers of properly paired alignments were randomly subsampled from the merged alignment of 150 hPSC samples.

Figure S3: Impacts of read depth on transcript assembly in simulated human pluripotent stem cell reads. A. Numbers of transcripts retrieved from high-depth simulated short reads. **B**. Coding to noncoding ratio for the transcripts assembled from high-depth simulated short-read samples. **C.** Sensitivity and precision of transcripts assembled from simulated short reads. **D**. The distribution of coding and noncoding transcripts in novel and GENCODE-annotated transcripts.

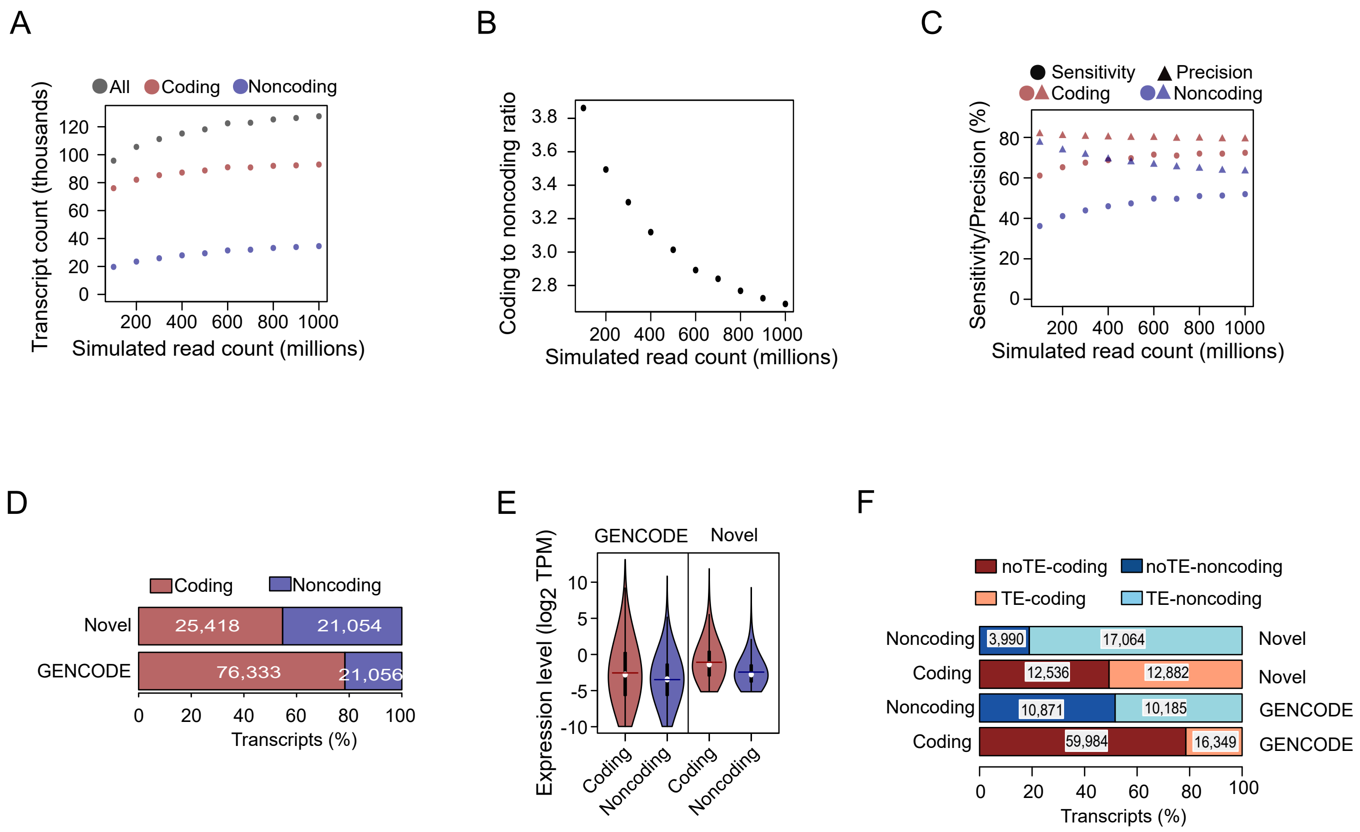

A
All
Coding
Noncoding
Transcript count (thousands)
Simulated read count (millions)
B
Coding to noncoding ratio
Simulated read count (millions)
C
Sensitivity
Precision
Coding
Noncoding
Sensitivity/Precision (%)
Simulated read count (millions)
D
Coding
Noncoding
Novel
GENCODE
25,418
21,054
76,333
21,056
Transcripts (%)
E
GENCODE
Novel
Expression level (log2 TPM)
Coding
Noncoding
Coding
Noncoding
F
noTE-coding
noTE-noncoding
TE-coding
TE-noncoding
Noncoding
Coding
Noncoding
Coding
3,990
17,064
12,536
12,882
10,871
10,185
59,984
16,349
Novel
Novel
GENCODE
GENCODE
Transcripts (%)

Figure S3

Transcript assembly from merged SRR597912 and SRR597895 reads. The transcripts were then classified into "novel" or GENCODE-annotated based on the presence in GENCODE annotation. Novel transcripts can be a new isoform of a GENCODE-annotated gene or transcripts that do not overlap any known GENCODE gene. **E**. The expression levels of coding and noncoding transcripts in GENCODE and novel transcript sets. **F**. Transposable element presence in coding and noncoding transcripts from GENCODE-annotated and novel sets.

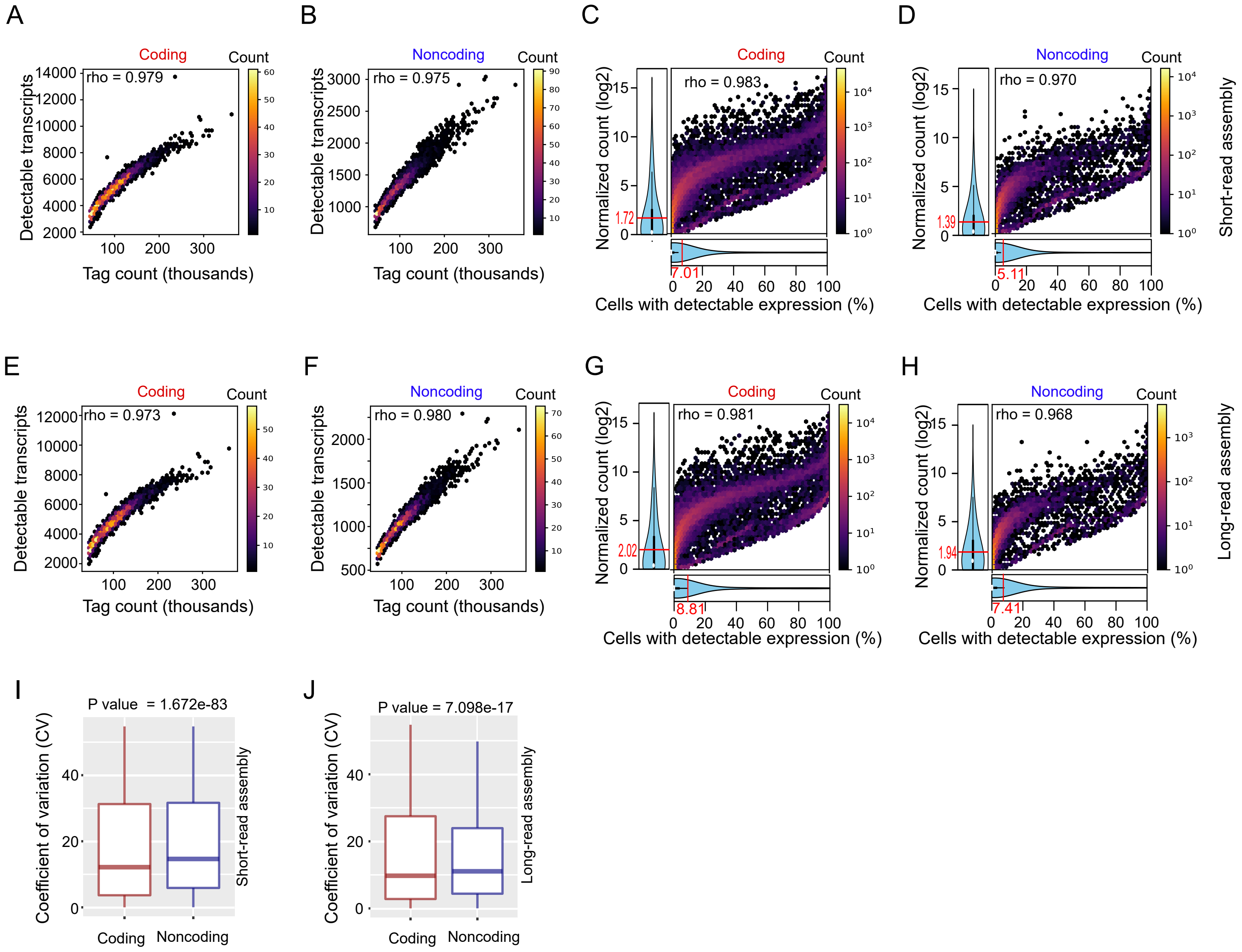

A
Coding
Count
rho = 0.979
Detectable transcripts
Tag count (thousands)
B
Noncoding
Count
rho = 0.975
Detectable transcripts
Tag count (thousands)
C
Coding
Count
rho = 0.983
Normalized count (log2)
1.72
7.01
Cells with detectable expression (%)
D
Noncoding
Count
rho = 0.970
Normalized count (log2)
1.39
5.11
Cells with detectable expression (%)
Short-read assembly
E
Coding
Count
rho = 0.973
Detectable transcripts
Tag count (thousands)
F
Noncoding
Count
rho = 0.980
Detectable transcripts
Tag count (thousands)
G
Coding
Count
rho = 0.981
Normalized count (log2)
2.02
8.81
Cells with detectable expression (%)
H
Noncoding
Count
rho = 0.968
Normalized count (log2)
1.94
7.41
Cells with detectable expression (%)
Long-read assembly
I
P value = 1.672e-83
Coefficient of variation (CV)
Short-read assembly
Coding
Noncoding
J
P value = 7.098e-17
Coefficient of variation (CV)
Long-read assembly
Coding
Noncoding

Figure S4

Figure S4: The heterogeneity of the assembled transcripts reveled by the single cell RNA-Seq data. The top 3000 cells with the highest number of tags were obtained from scRNA-seq alignment of c11/S0730 iPSC cell line. **Panels A-D** were obtained from the analyses of transcripts assembled from SRR597912 and SRR597895 short-read data, while **panels E-H** were obtained from the analyses of transcripts obtained from H9 long-read data assembly. Rho is the Spearman rank correlation coefficient. The numbers of detectable coding (**panel A**) and noncoding (**panel b**) transcripts assembled from the short-read data were positively correlated with the tag counts. The normalized expression levels of coding (**panel C**) and noncoding (**panel D**) transcripts assembled from the short-read data were positively correlated with the percent of cells with detectable expressions. The relationship between the numbers of detectable coding (**panel A**) and noncoding (**panel b**) transcripts assembled from the long-read data and the tag counts. The relationship between the normalized expression levels of coding (**panel C**) and noncoding (**panel D**) transcripts assembled from the long-read data and the percent of cells with detectable expressions. For **panels C-D and G-H**, distributions of the cells are shown in violin plots on the axes of the figures. The median values are shown in red fonts. Boxplots showing the coefficient of variations in the expressions for coding and noncoding transcripts in short-read (**panel I**) and long-read (**panel J)** data. The p values were computed with the Mann-Whitney tests.
